# Wide Bandwidth Class-S Power Amplifiers for Ultrasonic Devices

**DOI:** 10.3390/s20010290

**Published:** 2020-01-04

**Authors:** Kiheum You, Hojong Choi

**Affiliations:** Department of Medical IT Convergence Engineering, Kumoh National Institute of Technology, Gumi 39253, Korea; rlgma12@kumoh.ac.kr

**Keywords:** class-S power amplifier, wide band, power amplifier, ultrasound system

## Abstract

Wide bandwidth ultrasonic devices are a necessity in high-resolution ultrasonic systems. Therefore, constant output voltages need to be produced across the wide bandwidths of a power amplifier. We present the first design of a wide bandwidth class-S power amplifier for ultrasonic devices. The −6 dB bandwidth of the developed class-S power amplifier was measured at 125.07% at 20 MHz, thus, offering a wide bandwidth for ultrasonic devices. Pulse-echo measurement is a performance measurement method used to evaluate the performance of ultrasonic transducers, components, or systems. The pulse-echo signals were obtained using an ultrasonic transducer with designed power amplifiers. In the pulse-echo measurements, time and frequency analyses were conducted to evaluate the bandwidth flatness of the power amplifiers. The frequency range of the ultrasonic transducer was measured and compared when using the developed class-S and commercial class-A power amplifiers with the same output voltages. The class-S power amplifiers had a relatively flat bandwidth (109.7 mV at 17 MHz, 112.0 mV at 20 MHz, and 109.5 mV at 23 MHz). When the commercial class-A power amplifier was evaluated under the same conditions, an uneven bandwidth was recorded (110.6 mV at 17 MHz, 111.5 mV at 20 MHz, and 85.0 mV at 23 MHz). Thus, we demonstrated that the designed class-S power amplifiers could prove useful for ultrasonic devices with a wide frequency range.

## 1. Introduction

An increasing number of industrial fields are currently employing ultrasonic devices [1]. In the medical field, ultrasonic devices are used to examine the internal organs, and confirm the presence of the fetus and assess its movement [2,3,4,5]. In the industrial fields, these devices are used for underwater explorations, non-destructive testing, glass processing, in humidifiers, ultrasonic cleaners, and cell phones [1,6]. The ultrasonic signals from small-sized devices are very low, requiring a high voltage signal from the power amplifiers to properly trigger such devices [7,8].

**Figure 1:**
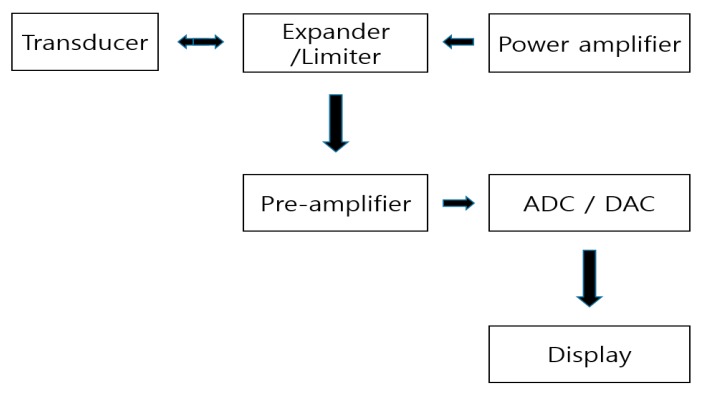
Block diagram of the ultrasonic system.

Figure 1 shows a simplified block diagram of an ultrasonic system [9,10]. In ultrasonic systems, the analog signal processing components (expander/limiter, power amplifier, and pre-amplifier) play a key role in determining the overall system performance [11]. The expander and limiter can reduce signal ring-down and block high voltage pulses, respectively [12]. The power amplifier, which is controlled by the digital-to-analog converter (DAC) and analog-to-digital converter (ADC), is used to achieve better energy transfer in electromechanical components, such as transducers [13]. The echo signals generated from the transducer are sent via the ADC to the display [11,14].

The power amplifiers used in communication systems are an important part of the transceiver (transmitter + receiver) systems [15]. In ultrasound (ultrasonic) systems, they also play an important role in operating the ultrasonic transducers by transmitting the high voltage or high-power signals in the desired frequency ranges [16,17]. A class-S type power amplifier was designed for ultrasonic systems to obtain a wide bandwidth in the high-frequency ranges, as the frequency characteristics of the ultrasonic transducers could be varied using a driving power amplifier [15]. Therefore, we aimed to cover wide frequency ranges of the ultrasonic transducer using the designed class-S power amplifiers.

To increase the bandwidth of the ultrasonic devices, the mechanical damping material is one of the design solution [18]. This material is useful to increase the bandwidth of the ultrasonic devices [19]. However, large size material, which has high acoustic impedance, could increase the bandwidth, and lower the sensitivity of the ultrasonic devices, because the mechanical damping absorb the part of the acoustic powers [20,21]. In addition, proper acoustic matching layers of the ultrasonic devices are used to increase the bandwidth, but also lower the sensitivity of the ultrasonic devices [22]. The electrical matching circuits are also useful to maximize energy transmission or increase the bandwidth of the ultrasonic systems by placing the matching circuits between the transducers and electronics [23,24]. However, this technique might also increase the pulse width, resulting in lowering the axial resolutions of the ultrasonic systems [25,26].

Several amplifiers have been developed for ultrasonic transducers. The power amplifiers driving the ultrasonic devices should have features, such as a high output voltage with efficient bandwidth, to obtain high image quality in the ultrasonic diagnostic systems [27,28,29]. However, these performances cannot be achieved simultaneously [30]. Therefore, the selection of power amplifier classes is important. We introduce the following characteristics based on the class of the power amplifiers. The class-A power amplifier has very high linearity with low power efficiency, while the class-B power amplifier has high linearity with moderately high power efficiency [31,32]. Therefore, a class-A power amplifier was proposed for high-frequency ultrasonic transducer [33]. Class-B amplifiers were implemented for an ultrasonic transducer [34]. The class-C power amplifier exhibits low linearity with high power efficiency; however, this has a narrow bandwidth [31,32]. The class-C power amplifier was introduced to increase the efficiency of portable ultrasound systems [35]. The class-D power amplifier offers high efficiency with low power loss, while the class–E power amplifier displays low linearity with high power efficiency, making this mode suitable for high power applications [31,32]. The class-D power amplifiers were used to increase the electrical power for high-intensity focused ultrasound transducers [36]. Class–D amplifiers were implemented for a 41 kHz Langevin sample transducer and high-power piezoelectric loads [37,38]. The class–E power amplifier offers efficient switching, which is useful in radio frequency applications [31,32]. High efficiency in the class-E power amplifier can be achieved by minimizing the power loss during the switching operation [31,32]. Class–E amplifiers were implemented for the 41.5 kHz piezoelectric transducers, which only functioned in certain narrow frequency ranges [39]. The class-S power amplifier has wide bandwidth to be used for various non-constant input waveforms, such as the load having low bandwidth or broad bandwidth [40].

**Figure 2:**
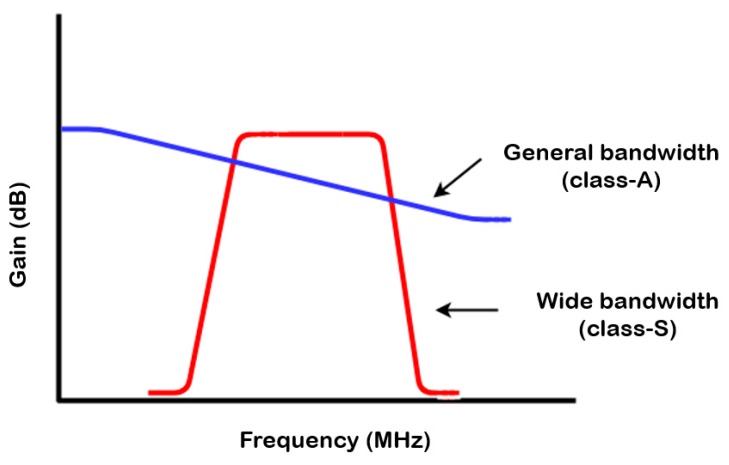
Performance comparison between the conventional class–A power amplifier and proposed class–S power amplifier at high frequencies.

High-resolution ultrasound equipment generally has a wide bandwidth from the ultrasonic devices, as the resolution depends on the bandwidth of these devices [7,41]. Therefore, as shown in Figure 2, a power amplifier that triggers the ultrasonic devices is preferred to obtain a wide bandwidth.

This paper is organized as follows. Section 2 analyzes the schematic and operating mechanism of a class–S power amplifier, while Section 3 shows the measurement results obtained using the class–S power amplifier. The outputs were verified over a wide input frequency range. Additionally, the pulse–echo signals were evaluated using ultrasonic transducers. Pulse–echo measurement is a performance measurement method used to evaluate the performance of ultrasonic transducers, components, or systems [42]. Time and frequency analyses were also conducted to evaluate the bandwidth flatness of the power amplifiers. Finally, Section 4 presents concluding remarks on the developed research.

## 2. Materials and Methods

In this study, a two-stage class–S power amplifier was designed. The first stage and second stage power amplifiers were combined to design a wider bandwidth class–S power amplifier as compared to a class-A power amplifier. In communications applications, the input signals of the class–S power amplifiers are used in combination with the modulated signals and adjacent narrow band frequency signals to cover relatively wide bandwidths, which are generally <50% [43]. In this paper, we do not use analog modulated signals. Therefore, the input signals in the wide frequency ranges (>50%) should be used to verify the ultrasonic devices.

### 2.1. Schematic of the Class–S Power Amplifier

**Figure 3:**
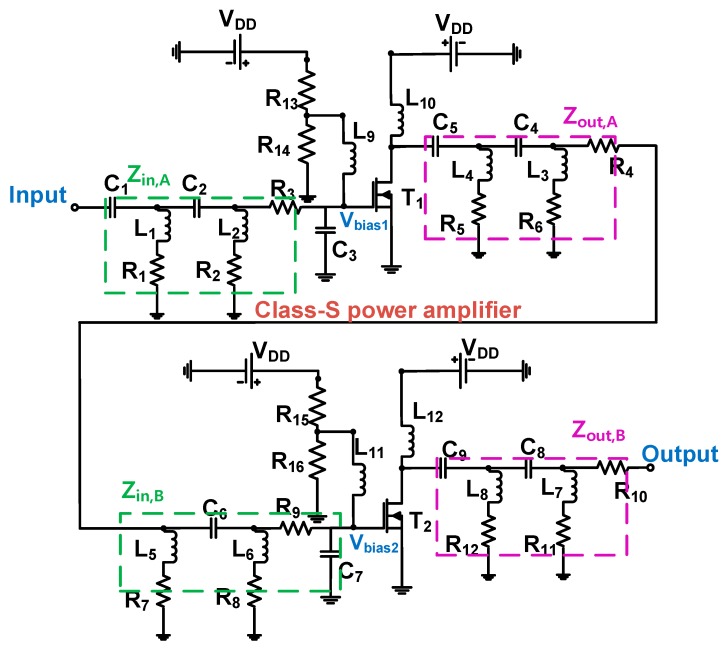
Schematic diagram of a class–S power amplifier with a resistor divider.

Figure 3 depicts the schematic circuit diagram of a two-stage class–S power amplifier. L_9_, L_10_, L_11_, and L_12_ are shunt choke inductors used to minimize the drop in the DC voltage. In the resistor divider, the bias voltages of the first and second stages were set to 4 V and 3 V, respectively. The input 50-Ω impedance matching circuit of the first stage was built using the capacitors C_1_ and C_2_, inductors L_1_, L_2_, and resistors R_1_, R_2_, R_3_, and C_3_. The output 50-Ω impedance matching circuit was constructed using the capacitors C_5_, C_4_, inductors L_4_, L_3_, and resistors R_5_, R_6_, and R_4_. The input 50-Ω impedance matching circuit of the second stage was built using the capacitor C_6_, inductors L_5_, L_6_, and resistors R_7_, R_8_, R_9_, and C_7_. The output 50-Ω impedance matching circuit was constructed using the capacitors C_9_, C_8_, inductors L_8_, L_7_, and resistors R_12_, R_11_, and R_10_. Additionally, an electrolytic capacitor (220 μF) and three capacitors (0.1 μF, 1000 pF, and 47 pF) were placed between the DC power supply and resistors (R_13_ and R_15_) to reduce noises from the DC power supply. A high-power metal-oxide-semiconductor field-effect transistor (MOSFET) was used for the power amplifier. The MOSFET (PD57006S–E, STMicroelectronics, Geneva, Switzerland) was selected as it can handle high levels of power, which has advantages, such as high switching speeds and a low gate drive power [31,44,45].

**Figure 4:**
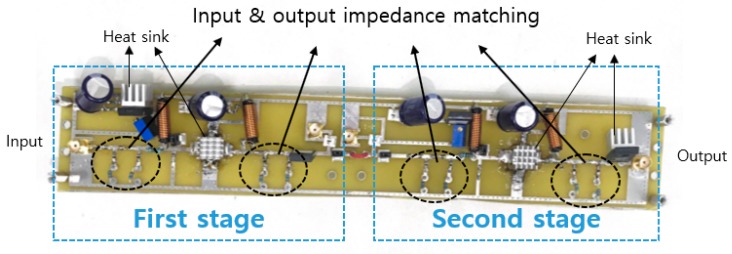
The printed circuit board (PCB) of a class–S power amplifier.

Figure 4 shows the fabricated printed circuit board (PCB) of a two-stage class-S power amplifier. The power amplifier is composed of power resistors, electrolytic capacitors, and high-power choke inductors to function appropriately in a high voltage environment. Additionally, a heat sink can be used with an external cooling fan to alleviate the heat generation problem associated with the current-heavy device. Table 1 shows the numerical values of the circuit element for class-S power amplifiers.

### 2.2. Analysis of the Class–S Power Amplifier

#### 2.2.1. First Stage Power Amplifier

The selected bias level allowed the transistor to operate as linearly as possible. The first stage of the power amplifier circuit uses 100% of the input signal for full linear operation, resulting in a conduction angle of 360° [46]. Therefore, the active devices are always in a conduction state. Additionally, the amplifiers in the first stage operate over the full range of input cycles, thus, minimizing distortion and providing high linearity. However, as the power supply current (I_DC_) is generated regardless of the input signal and active element, the efficiency is low, leading to increased heat and power consumption [32]. The drain current (i_D_) is given by [46]

(1) $$i_{D}=I_{\mathrm{DC}}+i_{\mathrm{rf}}\sin\omega_{o}t,$$

where I_DC_ is the bias current, i_rf_ is the signal amplitude of the drain current, and ω_o_ is the frequency of the signal. The output voltage (v_out_) can be obtained by multiplying the signal current (i_rf_) and load (R) [46].

(2) $$v_{\mathrm{out}}=-i_{\mathrm{rf}}\cdot R\sin\omega_{o}t.$$

The power (P_out_) of the signal delivered to the load, R is given by [46]

(3) $$P_{\mathrm{out}}=\frac{i_{\mathrm{rf}}{}^{2}\cdot R}{2}.$$

Additionally, i_rf_ is the same as I_DC_, so the DC power (P_DC_) is given by [46]

(4) $$P_{\mathrm{DC}}=I_{\mathrm{DC}}\cdot V_{\mathrm{DD}}=i_{\mathrm{rf}}\cdot V_{\mathrm{DD}}.$$

#### 2.2.2. Second Stage Power Amplifier

The operating point is a cutoff point assuming that the drain current (I_DQ_) = 0 and drain voltage (V_DQ_) = drain–source voltage (V_DS (cutoff)_) in the second stage of the power amplifier circuit. This ensures that a sinewave operates during the positive half cycle, with the negative half cycle equal to zero. Signal distortion is relatively high as a negative half cycle of the signal does not exist [46]. Additionally, the power loss increases with an increase in the conduction angle. However, the output efficiency is high in comparison with the first stage of the power amplifier. To minimize the above problem, symmetrical transistors and switching transistors are required, which also operate during the negative half cycles. However, this would not be suitable for the ultrasonic devices, which minimize/reduce the transformer ring-down caused by the symmetrical transistors. The drain current (i_D_) is given by [46]

(5) $$i_{D}=i_{\mathrm{rf}}\cdot \sin\omega_{o}t,\;\;\;\;i_{D}>0,$$

The output voltage (v_out_) and output power (P_out_) over half a cycle can be represented in terms of the load (R) [46]:

(6) $$v_{\mathrm{out}}=\frac{i_{\mathrm{rf}}}{2}\cdot R\sin\omega_{o}t,$$

(7) $$P_{\mathrm{out}}=\frac{v_{\mathrm{out}}{}^{2}}{2R},\;P_{\mathrm{out}(\max)}\;=\frac{v_{\mathrm{DD}}{}^{2}}{2R}.$$

Additionally, the maximum value of the output power (P_out(max)_) is related to the output voltage (v_out_) and drain voltage (V_DD_), so it can be expressed, as shown in Equation (7) [46]. The power (P_DC_) of the DC power supply is calculated based on the average value of the drain current (i_D.A_) and depends on the phase [46].

(8) iD.A=1T∫0T22VDDRsinωot dt=2VDDπR,

(9) $$P_{\mathrm{DC}}=\frac{2V_{\mathrm{DD}}{}^{2}}{\pi R}.$$

#### 2.2.3. Equivalent Circuit Analysis of a Two-Stage Power Amplifier

**Figure 5:**
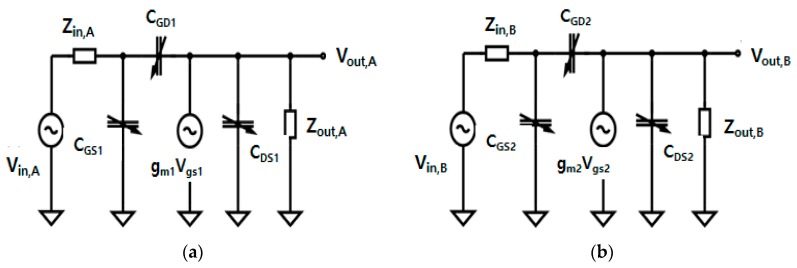
(**a**) Equivalent circuit of first stage, (**b**) equivalent circuit of second stage.

To simplify the equivalent circuit analysis, the small internal resistance and inductance values of each gate, drain, and source stage of the transistor in two-stage amplifier were ignored in the large signal nonlinear MOSFET model [46]. Figure 5 shows the equivalent circuit model of a simplified two-stage class–S power amplifier. The model consists of two stages, where V_in,A_ and V_in,B_ represent the voltage inputs of the first and second stages, respectively. V_out,A_ and V_out,B_ represent the voltage outputs of the first and second stages, respectively. Z_in,A_ and Z_out,B_ represent the impedance inputs of the first and second stages, respectively. Z_in,A_ and Z_out,B_ represent the impedance outputs of the first and second stages, respectively. C_GS1_ and C_GS2_ are the gate–source capacitors of the transistor. C_DS1_ and C_DS2_ are the drain–source capacitors of the transistor. C_GD1_ and C_GD2_ are the gate–drain capacitors of the transistor. C_GD1_, C_GD2_, C_GS1_, C_GS2_, C_DS1_, and C_DS2_ are the internal parasitic capacitors of the transistor, and their values depend on the applied gate source and gate drain voltages, and the load. The power gain of an amplifier is given by the ratio of the output power to the input power. In general, the equation for the power gain (P_Gain_) can be expressed as [46]:

(10) $$P_{\mathrm{Gain}}=\frac{V_{\mathrm{out},A}{}^{2}}{V_{\mathrm{in},A}{}^{2}}\cdot \frac{V_{\mathrm{out},B}{}^{2}}{V_{\mathrm{in},B}{}^{2}}(\frac{R_{\mathrm{in}}}{R_{\mathrm{out}}}).$$

R_in_ and R_out_ are the input and output impedances (Z) of 50-Ω in the function generator and oscilloscope. Thus, P_Gain_ can be expressed as follows [46]:

(11) $$P_{\mathrm{Gain}}=\frac{V_{\mathrm{out},A}{}^{2}}{V_{\mathrm{in},A}{}^{2}}\cdot \frac{V_{\mathrm{out},B}{}^{2}}{V_{\mathrm{in},B}{}^{2}}.$$

**Figure 6:**
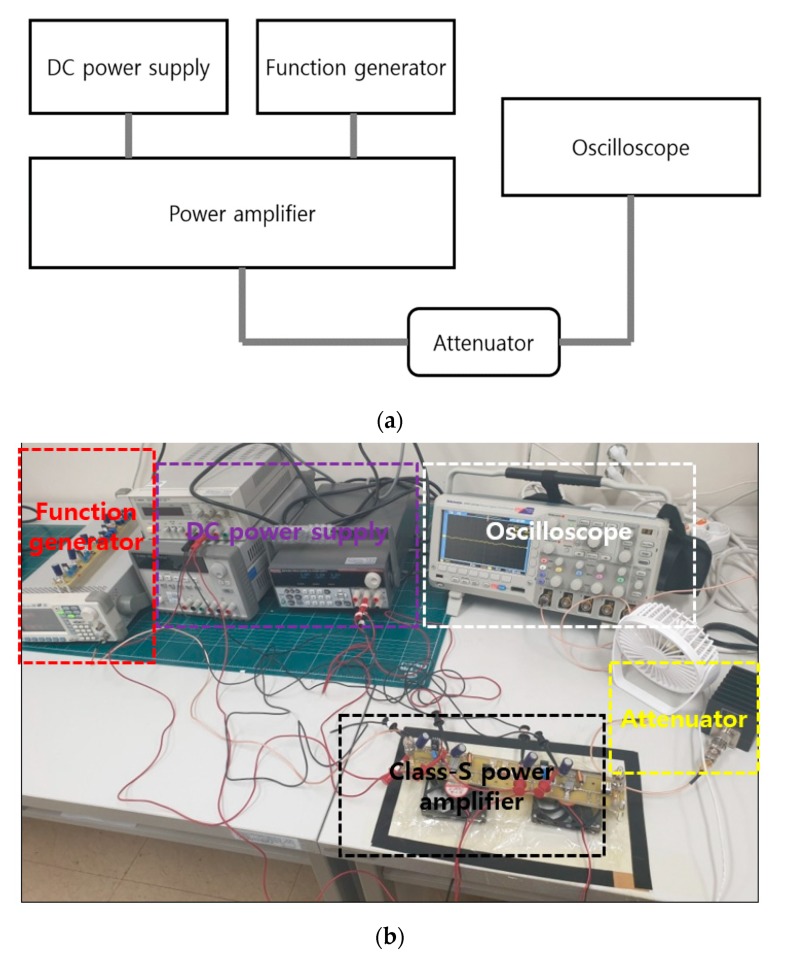
(**a**) Block diagram illustrating the performance measurement of a class–S power amplifier, (**b**) the measurement environment.

The power gains can be calculated using the equivalent circuits. The gain is obtained from the input and output voltages. The transfer functions ω_in_ and ω_out_ represent the input and output frequencies of each power amplifier stage [46]:

(12) $$\omega_{\mathrm{in}}=\frac{1}{Z_{\mathrm{in}}[C_{\mathrm{GS}}+(1+g_{m}Z_{\mathrm{out}})C_{\mathrm{GD}}]},$$

(13) $$\omega_{\mathrm{out}}=\frac{1}{Z_{\mathrm{out}}(C_{\mathrm{DB}}+C_{\mathrm{GD}})},$$

where ω is equal to 2πf_c_ and g_m_ represents the transconductance of the transistor. To obtain the transfer functions ω_in_ and ω_out_, the input and output impedances Z_in_ and Z_out_ are required. As depicted in Figure 6, the power amplifier is composed of two stages, so the input and output impedances of the first and second stages, Z_in,A,_ Z_out,A,_ Z_in,B_, and Z_out,B_, can be obtained as follows:

(14) $$Z_{\mathrm{in},A}=[\frac{1}{sC_{1}}+(sL_{1}+R_{1})\|\frac{1}{sC_{2}}]+[(sL_{2}+R_{2})\|R_{3}\|\frac{1}{sC_{3}}],$$

where s is equal to j2πf_c_, s^2^ is equal to −4πf_c_, f_c_ is the center frequency of the power amplifier, and Z_in,A_ is the input impedance of the first stage.

On the input side, the impedance can be calculated using the capacitor C_1_ connected in parallel with capacitor C_2_, inductor L_1_ and resistor R_1_, resistor R_3_ and capacitor C_3_ connected in parallel, and inductor L_2_ and resistor R_2_ present in the input matching circuit of the first stage.

(15) $$Z_{\mathrm{out},A}=[R_{4}+(sL_{3}+R_{6})\|\frac{1}{sC_{4}}]+[(sL_{4}+R_{5})\|\frac{1}{sC_{5}}],$$

where Z_out,A_ is the output impedance of the first stage. On the output side, the impedance can be calculated using the resistor R_4_ connected in parallel with capacitor C_4_, inductor L_3_ and resistor R_6_ connected in parallel with capacitor C_5_, and inductor L_4_ and resistor R_5_ present in the output matching circuit of the first stage.

(16) $$Z_{\mathrm{in},B}=[(R_{7}+sL_{5})\|\frac{1}{sC_{6}}]+[(R_{8}+sL_{6})\|R_{9}\|\frac{1}{sC_{7}}],$$

where Z_in,B_ is the input impedance of the second stage. On the input side, the impedance can be calculated using the capacitor C_6_ connected in parallel with inductor L_5_ and resistor R_7_, resistor R_9_ and capacitor C_7_ connected in parallel, and inductor L_6_ and resistor R_8_ present in the input matching circuit of the second stage.

(17) $$Z_{\mathrm{out},B}=[R_{10}+(sL_{7}+R_{11})\|\frac{1}{sC_{8}}]+[(sL_{8}+R_{12})\|\frac{1}{sC_{9}}],$$

where Z_out,B_ is the output impedance of the second stage. On the output side, the impedance was calculated using the resistor R_10_ connected in parallel with capacitor C_8_, inductor L_7_ and resistor R_11_ connected in parallel with capacitor C_9_, and inductor L_8_ and resistor R_12_ present in the output matching circuit of the second stage.

The input and output impedances Z_in,A_, Z_out,A_, Z_in,B_, and Z_out,B_ are obtained and then substituted into Equations (12) and (13). Therefore, the transfer functions of each stage can be obtained. The transfer function ω_in,A_ corresponding to the input side of the first stage is given by:

(18) $$\omega_{\mathrm{in},A}=\frac{\left[sC_{1}+s^{2}C_{1}C_{2}(sL_{1}+R_{1})]\cdot [R_{2}+R_{3}+sL_{2}+sC_{3}(R_{2}R_{3}+sL_{2}R_{3})]\cdot (s^{2}C_{4}L_{3}+sC_{4}R_{5}+1)\cdot (s^{2}C_{5}L_{4}+sR_{6}C_{5}+1\right)}{\begin{array}{l} \left[(R_{2}R_{3}+sL_{2}R_{3})\{sC_{1}+s^{2}C_{1}C_{2}(sL_{1}+R_{1})\}+(1+s^{2}L_{1}C_{2}+sC_{2}R_{1}+s^{2}C_{1}L_{1}+sC_{1}R_{1})\{R_{2}+R_{3}+sL_{2}+sC_{3}(R_{2}+R_{3}+sL_{2}R_{3}\right]\\ \cdot C_{\mathrm{GS}}(s^{2}C_{4}L_{3}+sC_{4}R_{5}+1)(s^{2}C_{5}L_{4}+sR_{6}C_{5}+1)+C_{\mathrm{GD}}[(s^{2}C_{4}L_{3}+sC_{4}R_{5}+1)(s^{2}C_{5}L_{4}+sR_{6}C_{5}+1)\\ +g_{m}\{(R_{6}+sL_{4})(s^{2}C_{5}L_{4}+sR_{5}C_{4}+1)+(s^{2}C_{5}L_{4}+sR_{6}C_{5}+1)(s^{2}C_{4}R_{4}R_{5}+R_{4}+R_{5}+sL_{3})\}] \end{array}}.$$

The transfer function ω_out,A_ corresponding to the output side of the first stage is given by:

(19) $$\omega_{\mathrm{out},A}=\frac{\left(s^{2}C_{4}L_{3}+sC_{4}R_{6}+1)(s^{2}C_{5}L_{4}+sR_{5}C_{5}+1\right)}{\left[(R_{5}+sL_{4})(s^{2}C_{4}L_{3}+sC_{4}R_{6}+1)+(s^{2}C_{5}L_{4}+sR_{5}C_{5}+1)(s^{2}C_{4}C_{3}R_{4}+sC_{4}R_{4}R_{6}+R_{4}+R_{6}+sL_{3})]\cdot (C_{\mathrm{DB}}+C_{\mathrm{GD}}\right)}.$$

The transfer function ω_in,B_ corresponding to the input side of the second stage can be expressed as:

(20) $$\omega_{\mathrm{in},B}=\frac{\left(s^{2}C_{6}L_{5}+sC_{6}R_{7}+1)\cdot [R_{8}+R_{9}+sL_{6}+sC_{7}(R_{8}R_{9}+sL_{6}R_{9})]\cdot [(s^{2}C_{8}L_{7}+sC_{8}R_{11}+1)(s^{2}C_{9}L_{8}+sR_{12}C_{9}+1)\right]}{\begin{array}{l} \left[(R_{7}+sL_{5})\{R_{8}+R_{9}+sL_{6}+sC_{7}(R_{8}R_{9}+sL_{6}R_{9})\}+(R_{8}R_{9}+sL_{6}R_{9})(s^{2}C_{6}L_{5}+sC_{6}R_{7}+1)\right]\\ \cdot [C_{\mathrm{GS}}(s^{2}C_{8}L_{7}+sC_{8}R_{11}+1)(s^{2}C_{9}L_{8}+sR_{12}C_{9}+1)+C_{\mathrm{GD}}\{(s^{2}C_{8}L_{7}+sC_{8}R_{11}+1)(s^{2}C_{9}L_{8}+sC_{9}R_{12}+1)\\ +g_{m}\{(R_{12}+sL_{8})(s^{2}C_{9}L_{8}+sRC+1)+(s^{2}C_{8}L_{7}+sC_{8}R_{11}+1)(s^{2}C_{8}L_{7}R_{10}+sC_{8}R_{10}R_{11}+R_{10}+R_{11}+sL_{7})\}] \end{array}}$$

The transfer function ω_out,B_ corresponding to the output side of the second stage is given by:

(21) $$\omega_{\mathrm{out},B}=\frac{\left(s^{2}C_{8}L_{7}+sC_{8}R_{11}+1)(s^{2}C_{9}L_{8}+sR_{12}C_{9}+1\right)}{\left[(R_{12}+sL_{8})(s^{2}C_{9}L_{8}+sR_{12}C_{9}+1)+(s^{2}C_{8}L_{7}+sR_{11}C_{8}+1)(s^{2}C_{8}L_{7}R_{10}+sC_{8}R_{10}R_{11}+R_{10}+R_{11}+sL_{7})]\cdot (C_{\mathrm{DB}}+C_{\mathrm{GD}}\right)},$$

where s = j2πf_c_ and s^2^= −4πf_c_. As the gain equals V_out_/V_in_, it can be represented as follows [46]:

(22) $$\frac{V_{\mathrm{out}}}{V_{\mathrm{in}}}(s)=\frac{-g_{m}Z_{\mathrm{out}}}{\left(1+\frac{s}{\omega_{\mathrm{in}}})(1+\frac{s}{\omega_{\mathrm{out}}}\right)}.$$

The transfer functions ω_in,A_, ω_out,A_, ω_in,B_, and ω_out,B_ are then substituted into Equation (22). This power amplifier circuit consists of two stages, and the total gain of V_out_/V_in_ of each stage can be obtained as follows. Using Equations (15), (18), and (19), the gain of the first stage is

(23) $$\frac{V_{\mathrm{out},A}}{V_{\mathrm{in},A}}(s)=\frac{-g_{m}(R_{5}+sL_{4})(s^{2}C_{4}L_{3}+sC_{4}R_{6}+1)+(s^{2}C_{5}L_{4}+sC_{5}R_{5}+1)(s^{2}C_{4}L_{3}R_{4}+sC_{4}R_{4}R_{6}+R_{4}+R_{6}+sL_{3})}{\left(s^{2}C_{4}L_{3}+sC_{4}R_{6}+1)\cdot (s^{2}C_{5}L_{4}+sR_{5}C_{5}+1)\cdot (1+\frac{s}{\omega_{\mathrm{in},A}})(1+\frac{s}{\omega_{\mathrm{out},A}}\right)}.$$

Using Equations (17), (20), and (21), the gain of the second stage is

(24) $$\frac{V_{\mathrm{out},B}}{V_{\mathrm{in},B}}(s)=\frac{-g_{m}(R_{12}+sL_{8})(s^{2}C_{9}L_{8}+sC_{9}R_{12}+1)+(s^{2}C_{8}L_{7}+sC_{8}R_{11}+1)(s^{2}C_{8}L_{7}R_{10}+sC_{8}R_{10}R_{11}+R_{10}+R_{11}+sL_{7})}{\left(s^{2}C_{8}L_{7}+sC_{8}R_{11}+1)(s^{2}C_{9}L_{8}+sR_{12}C_{9}+1)*(1+\frac{s}{\omega_{\mathrm{in},B}})(1+\frac{s}{\omega_{\mathrm{out},B}}\right)}.$$

The first and second stages are connected in series. Therefore, the gain (Gain) and power gain (P_Gain_) can be represented as:

(25) $$\mathrm{Gain}=\frac{V_{\mathrm{out},A}}{V_{\mathrm{in},A}}\cdot \frac{V_{\mathrm{out},B}}{V_{\mathrm{in},B}},\;P_{\mathrm{Gain}}=G{\mathrm{ain}}^{2}.$$

The basic gain function can be obtained from Equation (22). The final gain of the two-stage power amplifiers can be obtained by multiplying the gain of the first stage with the gain of the second stage, as shown in Equation (25). The power gain can be obtained by squaring the gain according to Equation (11).

However, this theoretical analysis may differ from the actual experimental results, as the simulations cannot accurately predict the signal distortion in power amplifier design, particularly in high-voltage environments [47]. Temperature parameters, heat generation, and external environmental factors can critically change the theoretical output performance of the amplifier. The transistors (MOSFETs) exhibit varied performance and accuracy depending on the high DC voltage and temperature [31]. In summary, the power amplifier analysis yields a theoretical value, which must be verified using actual measurement data to prove our proposed idea [47].

## 3. Results

### 3.1. Performance Analysis

Figure 6 shows the experimental measurement method employed in this study including the pulse-echo. The five–cycle sinewave was generated using a function generator, and the operating frequency and amplitude of the input voltage were increased. The power gain was obtained by measuring the output voltage of the power amplifier using an oscilloscope. Additionally, an attenuator was used to prevent overvoltage on the oscilloscope. Experiments were performed using a 40 dB attenuator with a 100× reduction in the signal amplitude. External coolers and heat sinks were used to alleviate the heat generation problems associated with the transistors and devices. The output voltage, power gain, and bandwidth were measured as a function of the input voltage and frequency.

**Figure 7:**
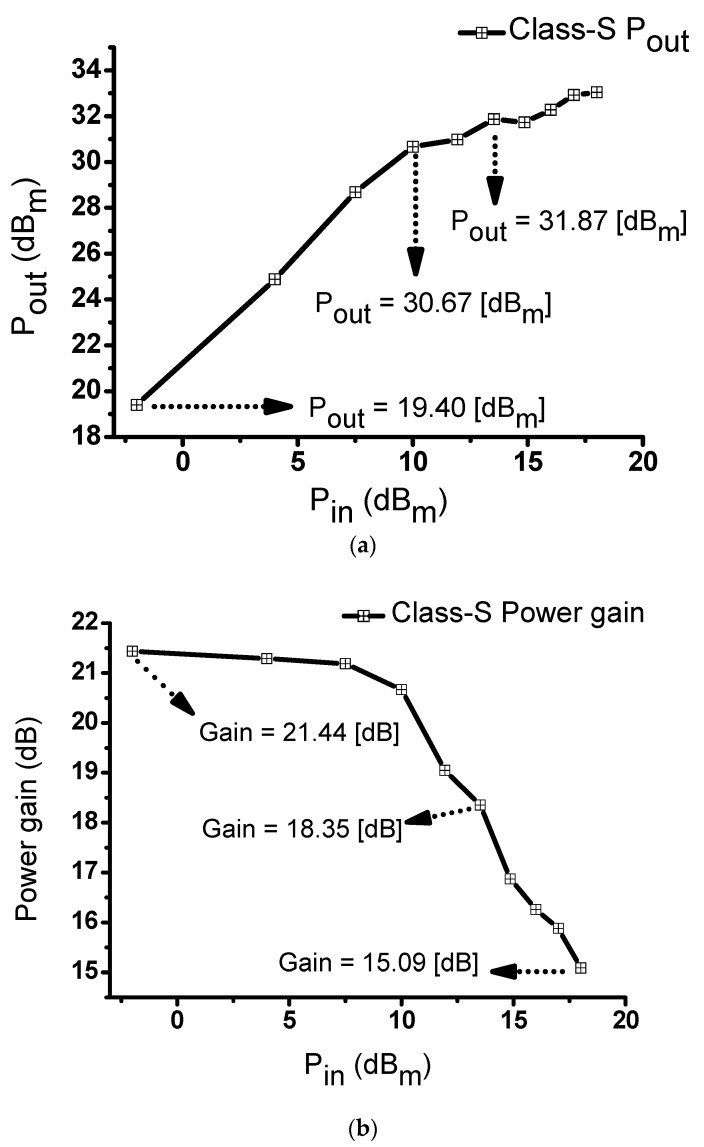

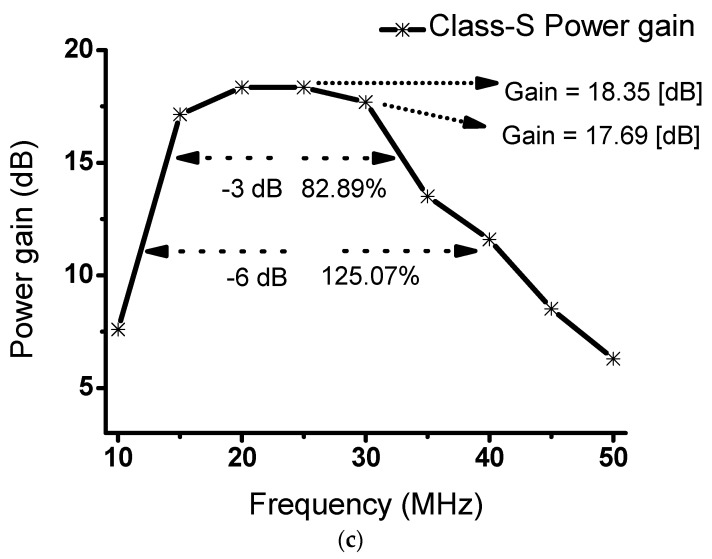
(**a**) Input power versus output power and (**b**) input power versus power gain observed in a class-S power amplifier with an input frequency of 20 MHz; (**c**) frequency versus output power of a class–S power amplifier with an input voltage of 3 V_P-P_.

Table 2 shows the simulated and measured results for the class-S power amplifier circuits. Figure 7 shows the measured results for the class-S power amplifiers. Figure 7a depicts the performance measurements when the input voltage of the class-S power amplifier was in the range of −2 to 18 dB_m_ at 20 MHz. At an input of 13.5 dB_m_, the DC current was 539 mA with an output of 31.87 dB_m_. At an input of 10 dB_m_, the measured output was 30.67 dB_m_, while at an input of −2 dB_m_, the measured output was 19.4 dB_m_. Figure 7b depicts the performance measurement when the input voltage of the class-S power amplifier was between −2 and 18 dB_m_ at 20 MHz. At an input of 13.5 dB_m_, the measured power gain was 18.35 dB. At the maximum input of 18 dB_m_, the output power was 33.04 dB_m_ with a DC current of 539 mA. The measured power gain was 15.09 dB. At an input of −2 dB_m_, the measured power gain was 21.44 dB. Figure 7c shows the power gain versus frequency at an input of 3 V_P-P_. The graph shows that we could achieve sufficient broad gain bandwidth from 15 MHz to 30 MHz. The maximum gain was 18.35 dB at 20 and 25 MHz, and an approximately uniform gain was measured from 17.15 dB at 15 MHz to 17.69 dB at 30 MHz. The −3 dB and −6 dB bandwidths were measured as 82.89% and 125.07%, respectively. Table 3 presents the measured current, output power, and power gain.

Table 3 shows the measured values from Figure 7a,b. The current, output power, and power gain varied according to the different inputs at 20 MHz.

Table 4 shows the measured values from Figure 7c, representing the measured power gain and output power at different frequencies.

### 3.2. Pulse-Echo Analysis

**Figure 8:**
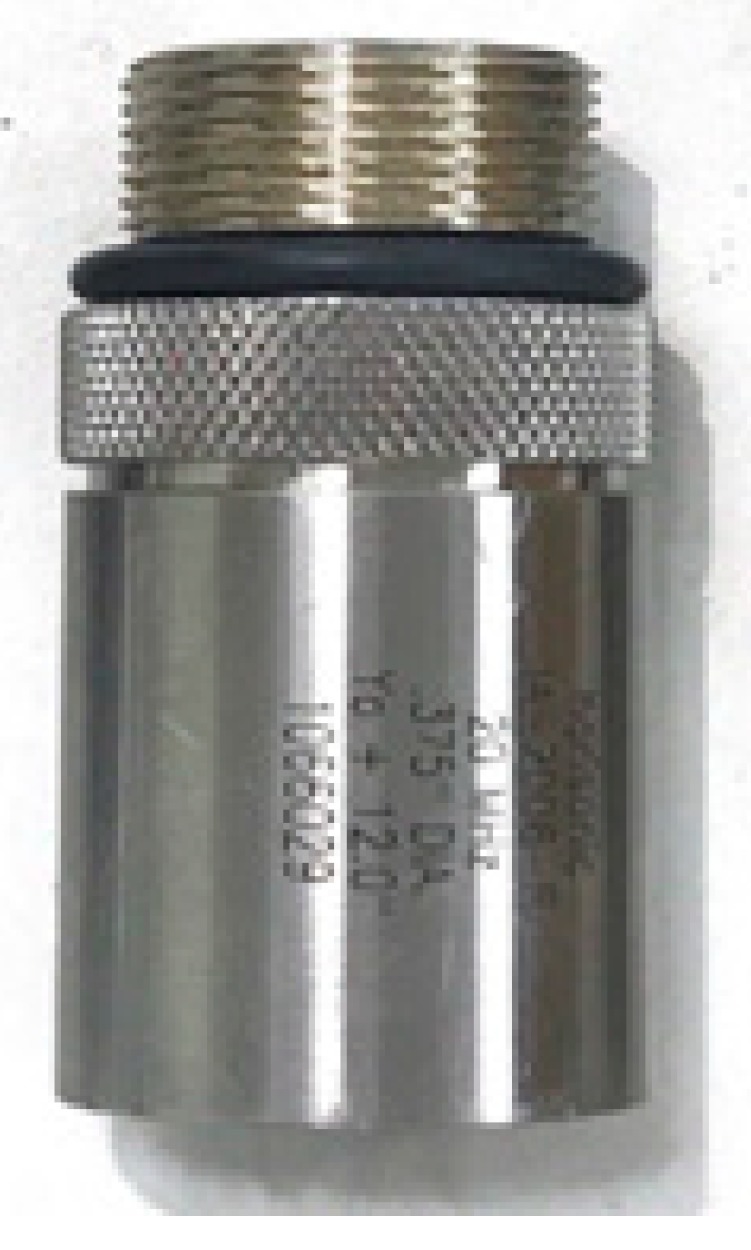
Twenty megahertz ultrasound transducer.

Pulse–echo measurement is a performance measurement method used to evaluate the performance of transducers, components, or systems [48,49]. In this method, an ultrasonic wave is generated by applying an electric signal to a transducer having a piezoelectric effect function. The electric signal from the transducer is then detected based on the wave reflected from the quartz target. In this study, the ultrasonic measurements were performed using a 20 MHz transducer, as shown in Figure 8. A single element transducer provided by Olympus (Shinjuku-ku, Tokyo, Japan) was employed for pulse-echo test. The distance between the transducer and target is 1.7 inch.

**Figure 9:**
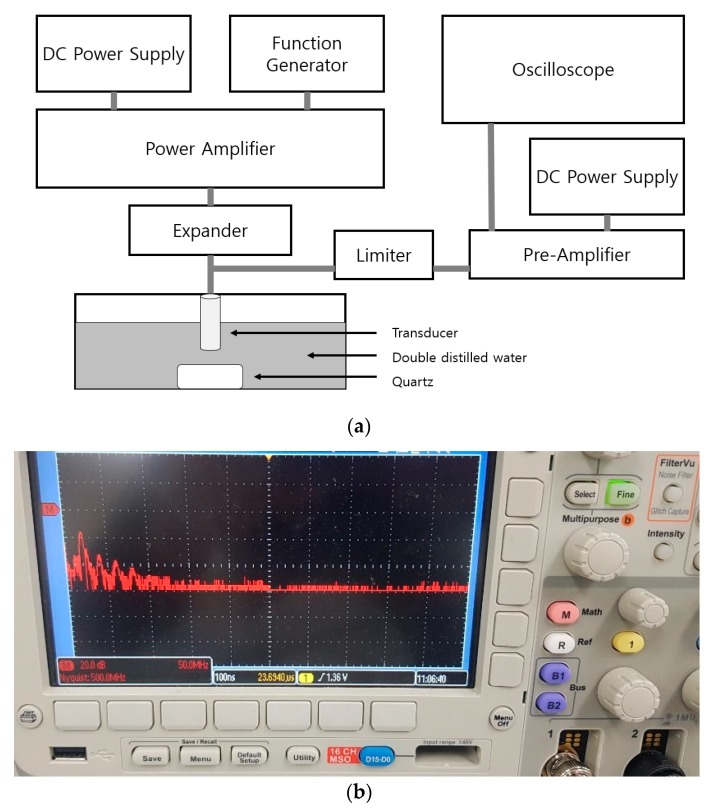
(**a**) Pulse-echo signal measurement setup for a class–S power amplifier and (**b**) enlarged pulse-echo spectrum in working status oscilloscope screen.

Figure 9a,b shows the procedure used to obtain the pulse-echo signal using a transducer in a water tank and enlarged pulse-echo spectrum in the oscilloscope. The quartz is the target object in the water tank, which contains double distilled water. The ultrasonic signals were measured via the transducer. A five-cycle sinusoidal waveform with a 20 MHz frequency and 3 V_P-P_ input voltage were generated using the function generator. The expanders were used to reduce the signal ring-down [50]. The limiter removed the high voltage signal, thus, protecting the pre–amplifier and oscilloscope [51]. The signal was amplified by the power amplifier and reflected by the quartz through the transducer [52]. The reflected ultrasonic signal was amplified by the pre–amplifier and then measured on the oscilloscope [53]. The echo signals and fast-Fourier transform (FFT) were measured/recorded from the power amplifiers.

**Figure 10:**
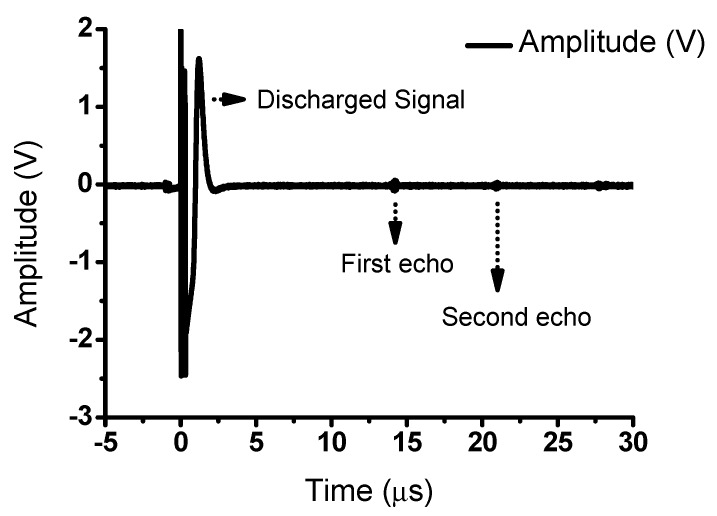
Pulse-echo experiment of a power amplifier.

Figure 10 shows the pulse-echo signal measurements obtained using a transducer, which indicates the performance of the ultrasonic power amplifier. A discharged signal that only passed through the pre–amplifier was detected, which was followed by the echo signal reflected by the quartz through the ultrasonic transducer. The quartz reflected 100% of the signal. In the field of ultrasonic, the echo signals are processed to obtain the output images. Therefore, the distortions in the echo signal were analyzed to obtain the total harmonic distortion (THD) since the signal distortions are related to image resolutions [54,55]. The THD represents the distortion caused by the unwanted harmonic components in the echo signal. The THD can be calculated using the following equation:

(26) $$\mathrm{THD}\frac{\sqrt{\mathrm{Second}.{\mathrm{Harmonic}}^{2}+\mathrm{Third}.{\mathrm{Harmonic}}^{2}}}{\mathrm{Fundamental}},$$

(27) THD (dB)=20·logTHD,

(28) THD (%)=100·THD.

**Figure 11:**
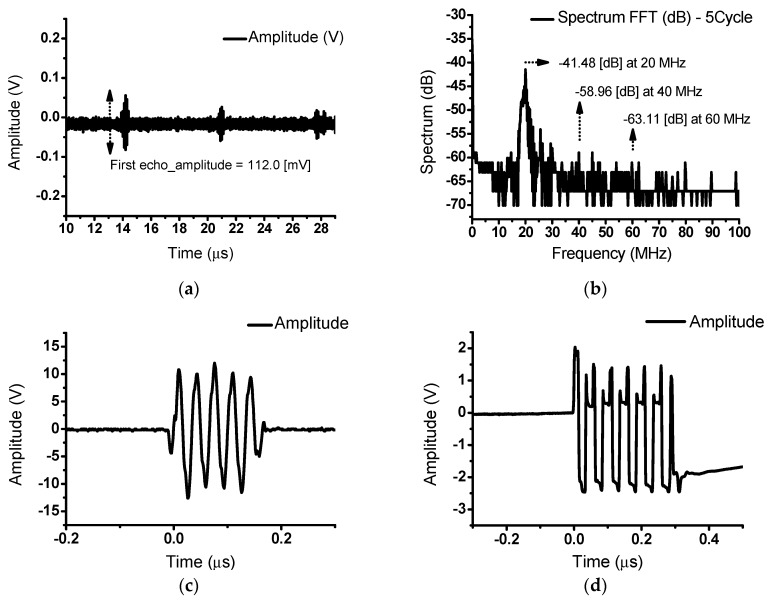
(**a**) Echo signals from a class-S power amplifier with an input voltage of 3 V_P-P_ and input frequency of 20 MHz; (**b**) FFT data of the echo signals; output signal of (**c**) the power amplifier and expander and (**d**) the limiter after the transducer.

Figure 11a shows the echo signal from a 20 MHz transducer. The measured echo signal was 112.0 mV for a 3 V_P-P_ and 20 MHz input. Figure 11b represents the spectrum data from the oscilloscope following application of the FFT. The measured fundamental signal, 2nd, and 3rd harmonic signals were −41.48 dB at 20 MHz, −58.96 dB at 40 MHz, and −63.11 dB at 60 MHz, respectively. The measured THD was −17.08 dB (13.99%). Figure 11c,d shows the output signal of the class-S power amplifier and expander circuit and the limiter after the transducer. These distorted signals were caused by each circuit element including the transducer device.

In the communication systems, the class-S power amplifiers use modulated signals, which consist of two adjacent frequency components. For the ultrasound instruments, each different frequency signal, as opposed to the modulated signals, was used to check the bandwidths of the echo signals. Therefore, the amplitude and bandwidth were verified in the wide frequency ranges covered by the transducer using a class-S power amplifier.

**Figure 12:**
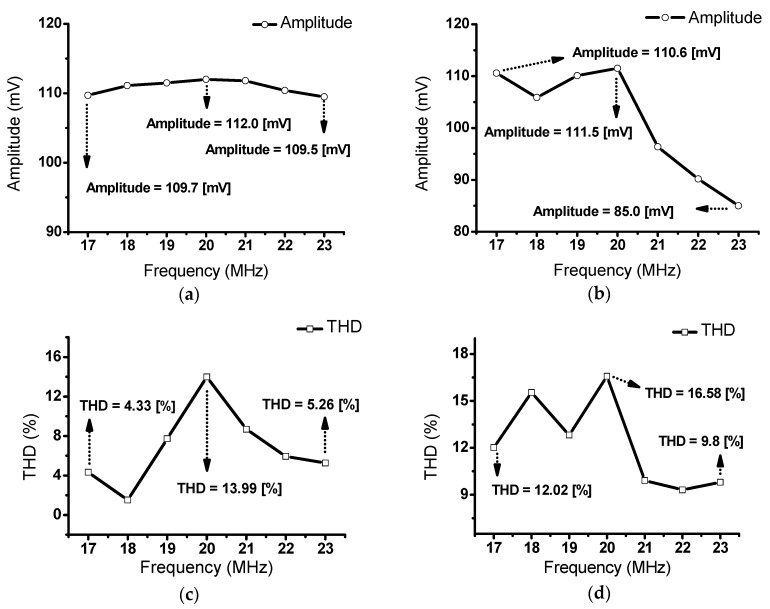

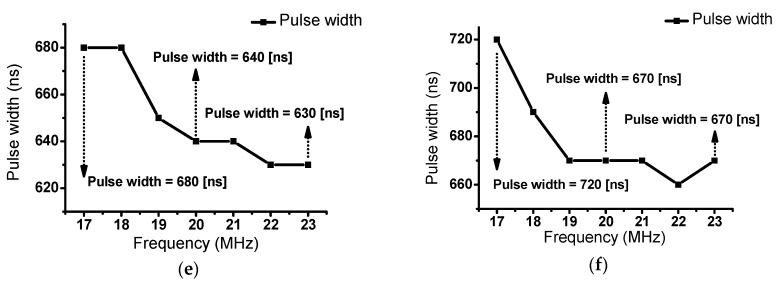
Echo signal bandwidth of a 20 MHz transducer when using a (**a**) class-S and (**b**) class-A power amplifiers with an input voltage of 3 V_P-P_; THD data when using (**c**) class-S and (**d**) class-A power amplifiers; pulse width data when using (**e**) class-S and (**f**) class-A power amplifiers.

Figure 12a shows the echo signal values corresponding to the different input frequencies (17–23 MHz) of the class-S power amplifier with a 20 MHz transducer. At an input of 3 V_P-P_, the recorded measurement data were 109.7 mV at 17 MHz, 112.0 mV at 20 MHz, and 109.5 mV at 23 MHz. The measurement results showed that the class–S power amplifiers could withstand wide bandwidths. Figure 12b shows the echo signal values corresponding to the different input frequencies (17–23 MHz) of a commercial 400 MHz class–A power amplifier (75A400, Amplifier Research, Souderton PA, USA). Measurements were performed under the same conditions as those employed in Figure 12. The measured echo signals were 110.6 mV at 17 MHz, 111.5 mV at 20 MHz, and 85.0 mV at 23 MHz. Differences in the amplitude and bandwidth can be observed when the results, shown in Figure 12a,b, are compared. The class–S power amplifiers could cover the 17 to 23 MHz frequency range. However, a class–A power amplifier could not exhibit a uniform signal, even though the class-A power amplifier had a flat gain in the wide frequency ranges. These measurement data demonstrate that the class–S power amplifiers could cover a wide bandwidth of the ultrasonic echo signal. As shown in Figure 12c,d, the measured THD value when using the class-S power amplifiers (13.99%) is lower than that when using the class-A power amplifier (16.58%) at 20 MHz. Therefore, these measurement data provides that the class-S power amplifier could offer lower echo signal distortions. Figure 12e,f shows the pulse widths of the echo signals. The pulse width is related to the axial resolution of the ultrasonic systems [56,57,58]. The pulse width of the echo signal when using the class-S power amplifier (640 ns) is shorter than that when using the class-A power amplifier (670 ns) at 20 MHz.

## 4. Conclusions

Wideband class–S power amplifiers are designed to cover the wide frequency ranges of ultrasonic devices. Various commercial amplifiers are available for ultrasonic applications. Therefore, we expect that wide bandwidth class–S power amplifiers will be increasingly drawing attention in high-frequency applications. In this study, a two-stage power amplifier was constructed to obtain a wide bandwidth for an ultrasonic transducer, which covered a wide frequency range. To verify the bandwidth of the developed class-S power amplifier, we measured the gain as a function of the operating frequency. For a 13.5 dB_m_ input at 20 MHz, the output power and power gain were 30.67 dB_m_ and 18.35 dB, respectively. The measured −3 dB and −6 dB bandwidths of the power amplifiers were at 82.89% and 125.07%, respectively. Additionally, the echo signal bandwidths of the wide band class–S and commercial class–A power amplifiers were compared using a 20 MHz transducer. The measured output voltages were 109.7 mV at 17 MHz, 112 mV at 20 MHz, and 109.5 mV at 23 MHz for the class–S power amplifier. When using the 20 MHz transducer, the class–S power amplifiers covered a bandwidth of 17–23 MHz, which was not achievable with the commercial class–A power amplifiers. Therefore, the designed class–S amplifier achieved a wider bandwidth when the echo signals were measured using transducers.

In current ultrasound instruments, many ultrasonic devices still require wide frequency ranges to obtain a high resolution. A wide bandwidth class–S power amplifier could be a potential candidate to produce transducers operating at a wide frequency range. Thus, the designed class–S power amplifier presented in this paper could potentially prove very helpful/useful for ultrasonic transducers, which require constant amplitudes at wide frequency ranges.

## Figures and Tables

**Table 1:** Numerical values of the circuit elements depicted in Figure 3.

Components	Values	Components	Values
C_1_	300 pF	L_1_, L_2_, L_3_, L_4_	1.2 µH
C_2_, C_5_,	1000 pF	L_5_, L_6_, L_7_, L_8_	1.2 µH
C_3_, C_7_	82 pF	L_9_, L_10_, L_11_, L_12_	1 µH
C_4_	170 pF	R_1_, R_2_, R_7_, R_8_	50 Ω
C_6_	20 pF	R_3_, R_9_	120 Ω
C_8_	60 pF	R_4_, R_10_	50 Ω
C_9_	200 pF	R_5_, R_6_	5 Ω
R_13_	320 Ω	R_11_, R_12_	3 Ω
R_14_, R_15_	110 Ω	R_15_	1.1 kΩ

**Table 2:** Simulated and measured results of class-S power amplifier.

	Simulated	Measured
Pin (dB_m_)	Pout (dB_m_)	Pout (dB_m_)
10	29.383	30.67
13.52	30.842	31.87
Pin (dB_m_)	Power gain (dB)	Power gain (dB)
10	19.383	18.35
13.52	19.404	15.09

**Table 3:** Class-S power amplifier measurements, corresponding to different inputs.

Pin (dB_m_)	Current (mA)	Pout (dB_m_)	Power Gain (dB)
−2	531	19.4	21.44
4	531	24.88	21.29
7.5	539	28.69	21.19
10	539	30.67	20.67
11.94	539	30.98	19.05
13.52	539	31.87	18.35
14.86	539	31.73	16.87
16	539	32.28	16.26
17	539	32.92	15.88
18	539	33.04	15.09

**Table 4:** Class-S power amplifier measurements, corresponding to different frequencies.

Frequency (MHz)	Pin (dB_m_)	Pout (dB_m_)	Power Gain (dB)
5	13.52	21.13	7.61
10	13.52	30.67	17.15
15	13.52	31.87	18.35
20	13.52	31.87	18.35
25	13.52	31.21	17.69
30	13.52	27.03	13.50
35	13.52	25.12	11.60
40	13.52	22.04	8.52
45	13.52	19.83	6.31
50	13.52	21.13	7.60
